# Shoulder impingement caused by distal clavicle osteochondroma

**DOI:** 10.1016/j.amsu.2021.103003

**Published:** 2021-11-03

**Authors:** Ismail Hamad Almogbil

**Affiliations:** Department of Surgery, Unaizah College of Medicine and Medical Science, Qassim University, Saudi Arabia

**Keywords:** Shoulder impingement, Osteochondroma, Exestotic bone

## Abstract

**Introduction:**

One of the most common causes of shoulder pain is impingement syndrome, several structural changes of acromion, coracoid process, coracohumeral ligament and acroimclavicular joint could cause it; however a rare extrinsic benign tumor such as osteochondromas could also lead to shoulder impingement which could be treated successfully by arthroscopy.

**Case report:**

We presented a successful arthroscopic resection of distal clavicle osteochondroma causing shoulder impingement in a 56-year-old male patient which was not responding to conservative management.

**Conclusion:**

Shoulder impingement could be caused by a very rare pathology such as osteochondromas. Sever shoulder pain and decrease of shoulder range of motion are common patient symptoms. Plain x-rays and MRI might be enough radiological investigation to reach up a diagnosis. Arthroscopic resection of such lesions considered as safe and effective management approach with low complication rates and recurrence rates.

## 1 Introduction

Neer described shoulder impingement in 1971 as a pathological reduction of the space underneath acromion process of the scapula [1].And it is considered one of the most common reasons for shoulder pain. Many factors may contribute to this pathology as classified by Ellman et al. intrinsic, extrinsic and secondary [2].

Any changes in morphology of the acromion, coracoid process, coracohumeral ligament and acroimclavicular joint could cause extrinsic subacromion impingement [3]. One of the rare causes of this pathology could be caused by benign tumors such as osteochondroma [4].

Shoulder impingement reach up to 65% of all shoulder pathologies and considered most common shoulder complaint [5].Osteochondromas arise from the metaphysis of long bones (distal femur, proximal tibia and proximal humerus) in up to 90% of cases, in other hand isolated flat bone (e.g. clavicle) osteochondromas are rare pathologies [6].

Osteochondroma may cause mechanical irritation and impingement if it originates from bones around the shoulder [7, 8]. We present here subacromial impingement with very rare etiology caused by exstotic bone from distal third of the clavicle which was treated successfully by arthroscopic resection.This case report has been written according to SCARE 2020 criteria [9].

## 2 Case report

F.N. a 56-year-old Saudi male patient, retired histopathologist, complaining of progressive left shoulder pain and discomfort with limitation of range of motion for the about 5 years. The patient was not known to have any medical problems, he denies any previous trauma on the same shoulder and he was not involved in any overhead or sports activities. His first visit to our clinic was in January 2021 for a second opinion, as he was diagnosed clinically with subacromial impingement in another clinic. All non-operative modalities were tried such as (NSAIDs, activity modification, physical therapy, and a subacromial corticosteroid injection) for 6 months without any improvement.

**Fig. 1:**
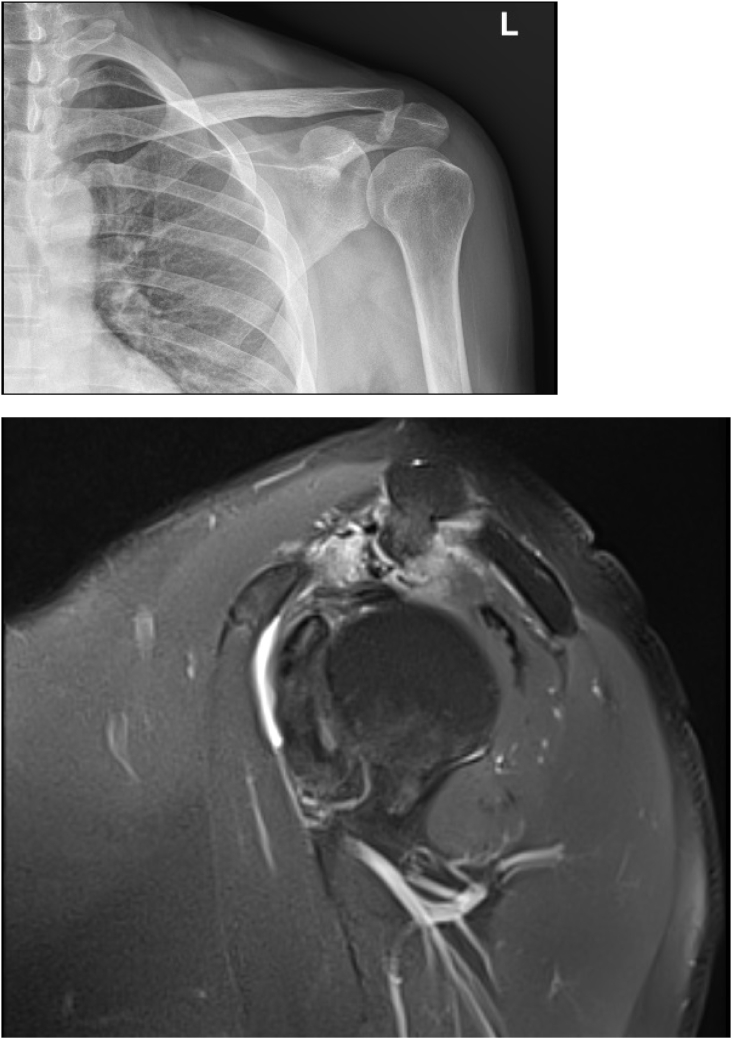
X-Ray Pre-operative showes distal clavicle exostotic bone and MRI revealed distal clavicle exostotic bone causing pressure effect on rotator cuff.

**Fig. 2:**
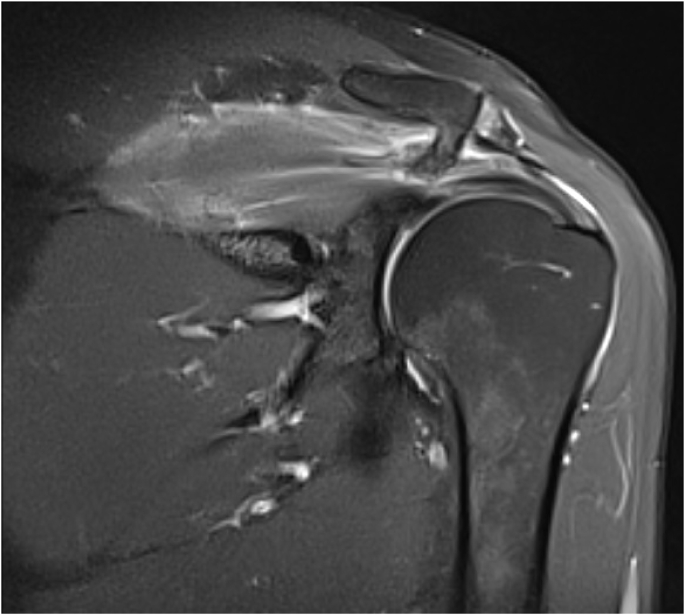
MRI revealed distal clavicle exostotic bone causing pressure effect on rotator cuff with cartilage cap.

X-rays showed an exostotic formation on the undersurface of the lateral third of the clavicle. Upon his clinical examination he had no muscle atrophy, no scapular dyskinesia or bony deformity. He had moderate tenderness over his acromioclavicular joint with pain provocation with shoulder internal rotation and abduction. ROM of his affected shoulder was restricted due to pain but full passive ROM. Special tests for impingement were positive both Neer and Hawkins. No other bone or joint pathology was noted. Plain radiographic images of his shoulder revealed a clear exostotic bony prominence projecting inferolateral and causing pressure effect on the rotator cuff (Fig. 1). Magnetic resonance (MR) confirmed pressure over the supraspinatus caused by an osseous formation from lateral third of the clavicle. A thin layer of cartilage was noted capping the exostotic bone suggestive of osteochondroma from distal clavicle (Fig. 2).

**Fig. 3:**
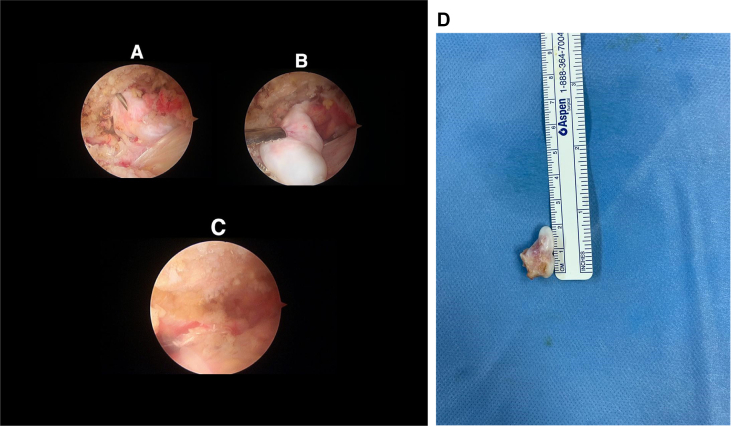
**A:** a spinal needle within AC joint and OC of distal clavicle. **B:** a grasper holding the OC after it was osteotomized. **C:** leveled undersurface of clavicle after removal of OC. *AC: Acromioclavicular joint, OC: Osteochondroma*. **D:** Gross picture of distal clavicle osteochondroma after excision.

**Fig. 4:**
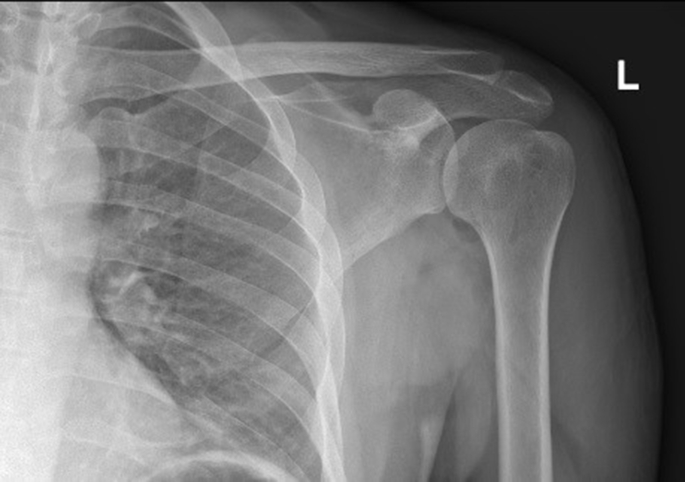
X-Ray showed post operative flat and complete removal of exostotic bone

After failure of non-operative modalities an arthroscopic excisional biopsy was indicated and accomplished. Glenohumeral diagnostic arthroscopy done first with no intra-articular pathology noted. Then subacromial space showed inflamed bursa with abundant bursal tissue. After a complete bursectomy rotator cuff tendons were intact and the exostotic bone noted as seen on the images protruding from lateral third of the clavicle (Fig. 3 A). With a small osteotome the exostotic bone was removed as one piece measured (2cm in length) (Fig. 3B-D). A histopathology report confirmed the diagnosis of benign osteochondroma. Post-operative rehabilitation consists of arm sling for 7 days. Then mobilization was commenced under the supervision of a physiotherapist. At three months post-operative he had complete resolution of symptoms with full pain free range of motion. No recurrence was seen on radiographs 6 months after surgery (Fig. 4).

## 3 Discussion

Benign bone tumors are frequently seen in orthopedics practice like osteochondromas (OC) which is up to 50% of prevalence [10]. Different types of OC have been described in literature most of these predominate in male and younger than 20 years of age [11]. Osteochondromas are described as bony projection with cartilage cap over the surface of the bone [12]. Histologically the cap is composed of hyaline cartilage resembles that of a normal growth plate [13]. These lesions cause pressure effect over the rotator cuff and subacromial bursa which manifest as shoulder pain and impingement with possibility of reduction in shoulder range of motion. Early routing imaging is advocated in patient with mechanical shoulder pain to help for early diagnosis [14]. X-rays are with low cost and gives enough information to rule out external impingement causes and help early diagnosis and management.

Smith et al. described a Clavicular OCs as rare pathology, accounts for 0.2–0.5% of all solitary OCs [15].Only Few cases of distal clavicle OCs have been published in literature [16,17]. Most of the cases were treated by open approach to excise the lesion. Messinese et [17] al, Kim et al. [18] and Simon Thomas et al. [19] Used all arthroscopic excision of the lesions without the need for open surgery by which they avoid larger incisions, no deltoid detachment and lesser infection rate. With advanced development of new arthroscopic techniques and instruments, we believe arthroscopic excision of such lesions gives better surgical outcomes, surgically less invasive, faster recovery, and shorted days of admission than open surgery. Insufficient lesion excision could give up to 2% recurrences rate in open surgical excision [10], however it is not yet reported after arthroscopic excision.

## 4 Conclusion

Although OCs considered a rare cause of shoulder impingement however, we have to have low threshold to request routine radiographic investigation to exclude extrinsic causes of shoulder impingement. Arthroscopic management of OCs is safe and effective approach when several factors such as lesion size, site and surgeon experience are considered.

## Ethical approval

Written informed consent for publication of their clinical details and/or clinical images was obtained from the patient. A copy of the written consent is available for review by the Editor-in-Chief of this journal on request.

## Sources of funding

No funding to be declared.

## Author contribution

Ismail Almogbil: written the paper and operating surgeon.

## Registration of research studies

1. Name of the registry:
2. Unique Identifying number or registration ID:
3. Hyperlink to your specific registration (must be publicly accessible and will be checked):

## Guarantor

Professor Abdulaziz AlAhaideb.

## Provenance and peer review

Not commissioned, externally peer-reviewed.

## Consent

Written informed consent for publication of their clinical details and/or clinical images was obtained from the patient. A copy of the written consent is available for review by the Editor-in-Chief of this journal on request.

## Declaration of competing interest

The authors declare having no conflicts of interest for this article.
